# Complete mitochondrial genome of little ringed plover *Charadrius dubius* (Charadriiformes, Charadriidae)

**DOI:** 10.1080/23802359.2022.2134746

**Published:** 2022-11-04

**Authors:** Dong Yun Lee, Seung Jin Roh, Sung Hyun Kim, Tae Won Jung, Dong June Lee, Hyun Kyong Kim, Ji Hwa Jung, Sook-Young Cho, Yun Jung Kim, Ji Won Kook, Ha Cheol Sung, Ju Hyun Lee, Woo Yuel Kim

**Affiliations:** a School of Biological Sciences and Biotechnology Graduate School, Chonnam National University, Gwangju, South Korea; b Division of Zoology, Honam National Institute of Biological Resources, Mokpo, South Korea; c Department of Biological Sciences, College of Natural Sciences, Chonnam National University, Gwangju, South Korea; d Research Center of Ecomimetics, Chonnam National University, Gwangju, South Korea

**Keywords:** Mitochondrial genome, Charadriiformes, *Charadrius dubius*, little ringed plover

## Abstract

This study encoded the complete mitochondrial genomic sequence of the little ringed plover *Charadrius dubius*. The mitochondrial genome has a total length of 16,864 bp, consisting of 13 protein-coding genes, 22 tRNA genes, two rRNA genes, and a control region. The nucleotide composition was 23.8% T, 31.6% A, 30.8% C, and 13.8% G. This study provides the basic information on the mitogenome of *C. dubius* and supports the understanding of mitogenomic information and its phylogenetic relationship within Charadriiformes.

The little ringed plover, *Charadrius dubius* Scopoli, 1786 has a black mask around its face with yellow eye rings and belongs to the family Charadriidae (Carter and Rogers 1998). This species is widely distributed from Africa to Eurasia, with its breeding grounds from Europe and India to East Asia, including the Korean Peninsula (Colwell and Haig 2019). According to previous studies about the taxonomy of plovers, *Charadrius* Linnaeus, 1785 were classified into the two existing major clades (i.e. CRD I and CRD II); *C. dubius* was also included in CRD I groups (Dos Remedios et al. 2015). In this study, we sequenced the complete mitochondrial genome of *C. dubius* and conducted phylogenetic analysis with the related taxa, which enhanced the basic genetic information of the genus *Charadrius*. We also conducted phylogenetic analysis with related taxa by sequencing the complete mitochondrial genome of this species.

We captured an individual of *C. dubius* from the Mangeoyong estuary (N 35°52′51.5″, E 126°40′58.9″) in Gunsan-si, Jellabuk-do, South Korea, using a funnel trap on 30 June 2021; this individual was deposited in the Chonnam National University, Gwangju, South Korea (Voucher storage: Chonnam National University; voucher number: MLR-1; the person in charge of collection was DY Lee; email: ghjkl833@naver.com). The blood sample was collected from the capillary vessel of the captured bird using a micro syringe, and the total genomic DNA was extracted using DNeasy Blood and Tissue kit (Qiagen, Valencia, CA) following the protocol of the manufacturer. Blood and DNA samples were stored in a freezer at −20 °C.

The complete mitochondrial genome of *C. dubius* was sequenced using Illumina NovaSeq 6000 (Macrogen, Inc., Seoul, South Korea). A total of 5,786,919,729 read bases of filtered data were analyzed to generate 38,416,380 reads of sequence; these were then assembled in Geneious Prime (Kearse et al. 2012). Gene annotation was accomplished and circularity was checked using the MITOS web server (Bernt et al. 2013, http://mitos.bioinf.uni-leipzig.de/), and secondary structures of tRNA genes were analyzed by comparing them to the nucleotide sequences of other bird species’ tRNA sequences.

The complete mitochondrial genome sequence of *C. dubius* has a total length of 16,864 bp, which was close to the other reported mitogenomes sizes of Charadrii, which range from 16,791 bp to 17,378 bp; minimal length variation was observed in PCGs, tRNAs, and rRNAs (Chen et al. 2018). The mitogenome contains 23.8% T, 31.6% A, 30.8% C, and 13.8% G, which showed a high A + T content; overall AT content of *C. dubius* mitogenome was 55.3%, which is consistent with previous Charadrii mitogenome studies (Li et al. 2014; Hu et al. 2017; Chen et al. 2018). All protein coding genes have ATN as their start codon, except *COX1* and *ND5*, which use the start codon GTG. The stop codon T– only appeared in the *COXIII*, *ND2*, and *ND4*. A truncated stop codon may be completed through the poly-adenylation of the 3′-end of the mRNA via post-transcriptional processes (Lavrov et al. 2002; Chen et al. 2018). Additionally, an extra nucleotide (C: cytosine) was present at the position 174 of the *ND3* gene for 16 species of Charadrii (Mindell et al. 1998; Chen et al. 2018); the full length of the D-loop region is 1301 bp.

After removing the termination codon and indels, total length 11,397 bp of the protein-coding genes was used for phylogenetic analysis of 13 species. The tree was constructed with maximum-likelihood method using RAxML version 8.1.2 (Stamatakis 2014) with 1000 bootstrap replications. *Haematopus ater* Vieillot, 1825 and *H. ostralegus* Linnaeus, 1758 from the family Haematopodidae were used as the outgroup species in this study. This analysis facilitated the construction of a robust phylogenetic tree with high supports for all nodes (see Figure 1).

**Figure 1.**
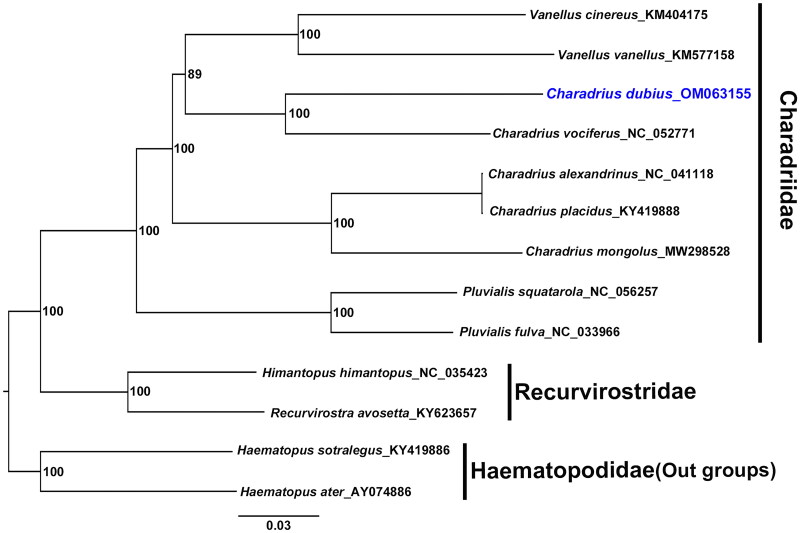
Phylogenetic tree of *C. dubius* (blue text) with eight other species in Charadriidae, two species in Recurvirostridae, and two species in Haematopodidae. This tree is based on 13 protein-coding genes, constructed using the maximum-likelihood (ML) method. Numbers on each branch indicate the bootstrap support value for 1000 replicates.

Phylogenetic analysis showed that family Charadriidae is a monophyly group, but the genus *Charadrius* is not. Moreover, *C. dubius* was found to be more closely related to *C. vociferous* Linnaeus, 1758, *Vanellus vanellus* (Linnaeus, 1758), and *V. cinereus* (Blyth, 1842) than it was to *C. placidus* Gray & Gray, 1863, which was previously hypothesized to be its closest relative on the molecular phylogenetic tree (Dos Remedios et al. 2015; Colwell and Haig 2019). Further research is thus needed to thoroughly understand the morphological characters and conduct in-depth molecular phylogenetic analyses of *Charadrius* and *Vanellus* genera.

The results of this study present the complete genetic information of the little ringed plover and propose avenues for future research to improve scientific understanding of the phylogenetic relationships within various species of plovers.

## Ethical approval

Permission for the capture and collection of wild animals was granted by the Gunsan City Environment Policy Division [No.-15746 (2021.06.29) 2021-6]. The study species used in this research are not included in the IUCN red list and individuals were not collected from protected areas. All procedures conducted to produce and publish this article were conducted in compliance with the regulations of the Honam National institute of Biological Resources (HNIBR).

## Data Availability

Genome sequence data that support the findings of this study are openly available from the NCBI GenBank at https://www.ncbi.nlm.nih.gov/, under accession no. OM063155. The associated BioProject, SRA, and Bio-Sample numbers are PRJNA803930, SRP358595, and SAMN25690482, respectively.
